# Mitochondrial epigenetics in aging and cardiovascular diseases

**DOI:** 10.3389/fcvm.2023.1204483

**Published:** 2023-07-13

**Authors:** Alessia Mongelli, Alessandro Mengozzi, Martin Geiger, Era Gorica, Shafeeq Ahmed Mohammed, Francesco Paneni, Frank Ruschitzka, Sarah Costantino

**Affiliations:** ^1^ Center for Translational and Experimental Cardiology (CTEC), Department of Cardiology, Zurich University Hospital and University of Zürich, Zurich, Switzerland; ^2^ Department of Cardiology, University Heart Center, University Hospital Zurich, University of Zurich, Zurich, Switzerland.

**Keywords:** mitoepigenetics, mtDNA, ncRNAs, mitochondria, aging, cardiovascular diseases, methylation

## Abstract

Mitochondria are cellular organelles which generate adenosine triphosphate (ATP) molecules for the maintenance of cellular energy through the oxidative phosphorylation. They also regulate a variety of cellular processes including apoptosis and metabolism. Of interest, the inner part of mitochondria—the mitochondrial matrix—contains a circular molecule of DNA (mtDNA) characterised by its own transcriptional machinery. As with genomic DNA, mtDNA may also undergo nucleotide mutations that have been shown to be responsible for mitochondrial dysfunction. During physiological aging, the mitochondrial membrane potential declines and associates with enhanced mitophagy to avoid the accumulation of damaged organelles. Moreover, if the dysfunctional mitochondria are not properly cleared, this could lead to cellular dysfunction and subsequent development of several comorbidities such as cardiovascular diseases (CVDs), diabetes, respiratory and cardiovascular diseases as well as inflammatory disorders and psychiatric diseases. As reported for genomic DNA, mtDNA is also amenable to chemical modifications, namely DNA methylation. Changes in mtDNA methylation have shown to be associated with altered transcriptional programs and mitochondrial dysfunction during aging. In addition, other epigenetic signals have been observed in mitochondria, in particular the interaction between mtDNA methylation and non-coding RNAs. Mitoepigenetic modifications are also involved in the pathogenesis of CVDs where oxygen chain disruption, mitochondrial fission, and ROS formation alter cardiac energy metabolism leading to hypertrophy, hypertension, heart failure and ischemia/reperfusion injury. In the present review, we summarize current evidence on the growing importance of epigenetic changes as modulator of mitochondrial function in aging. A better understanding of the mitochondrial epigenetic landscape may pave the way for personalized therapies to prevent age-related diseases.

## Introduction

Mitochondria are double membrane organelles which are actively involved in a multitude of cellular activities such as energy production in the form of adenosine triphosphate (ATP), intracellular Ca^2+^ signalling, generation of reactive oxygen species (ROS), and catalysis of metabolites (1)⁠. Physiologically, the high plasticity of mitochondria makes them able to respond rapidly to cellular metabolic demands, such as during physical activity or fasting (1)⁠. Mitochondria are dynamic organelles that constantly alter their shape, oscillating between two opposing processes, fission and fusion, in response to different stimuli (1, 2).

In healthy individuals, these processes are well balanced. In fact, if the organism requires more energy, two mitochondria fuse together. To increase the amount of ATP, the cell transcribes the nuclear genes encoding mitochondrial proteins (NuGEMPs) which in turn promote the activity of the outer mitochondrial membrane (OMM) proteins, Mitofusin 1 and 2 (Mnf1/2) and the inner mitochondrial membrane (IMM) protein, Optic Atrophy Protein 1 (Opa1) (3)⁠.

On the other hand, under conditions of reduced energy demand or in the presence of dysfunctional mitochondria, such as during a sedentary lifestyle or aging, the fission mechanism (the removal of a mitochondria) is enhanced (1). This mechanism, triggered by the reduction of inner membrane potential or by high ROS production, induces the interaction among dynamin like 1 (Drp1), fission mitochondrial 1 (Fis1) and mitochondrial fission factor (Mff) (4). In physiological conditions, the unneeded or damaged mitochondrial fragments are cleared by the autophagosome (mitophagy process), while the accumulation of damaged mitochondria is strictly linked to the exacerbation of neurodegenerative and cardiovascular diseases, cancer and inflammation (5–8).

These mechanisms are also influenced by mitochondrial DNA (mtDNA). In fact, cells contain 100–10,000 copies of mtDNA in proportion to energy request (9)⁠. Despite some similarities with the genomic DNA, the mtDNA has specific features. First, the hereditary is not Mendelian because all mitochondria are transmitted by uniparental model (maternal hereditament). Second, the mtDNA is a circular covalently closed double stranded DNA with a length of 16.5 kbs in human (10)⁠. To distinguish the two strands, one is named “heavy” (the sense, which is purine rich), and the other is named “light” (the antisense, which contains high amounts of pyrimidine). The mtDNA has 37 genes, which encode 13 polypeptides involved into the oxidative phosphorylation, 2 rRNAs and 22 tRNAs (11)⁠. Third, similar to genomic DNA, the mtDNA assumes a secondary structure that regulates the genes transcription and the synthesis of new mtDNA molecules (12)⁠. This upper level of mitochondrial gene regulation has been named as “*mitoepigenetics*” (13–15)⁠.

## Features of mitochondrial epigenetics (mitoepigenetics)

Unlike genomic DNA, mtDNA is not packed into nucleosomes but is organized into protein complexes in which mtDNA is bound to mitochondrial transcription factor A (TFAM) to form nucleoids measuring 100 nm in diameter (16, 17). Despite its name, TFAM is not the main transcriptional player; in fact, mitochondrial RNA polymerase (POLRMT) and mitochondrial transcription factor B2 (TFB2M) are mainly involved in mitochondrial mRNA synthesis (18). However, it is essential for the maintenance, expression and transmission of mitochondrial DNA (mtDNA). Interestingly, post-translational modifications (PTMs) of TFAM play an important role in its affinity for mtDNA. In particular, acetylation in lysine (K62, K76, K111 and K118) or phosphorylation (S55 and/or S56) in serine can fine-tune TFAM-DNA binding affinity (19).

Specifically, acetylation of TFAM at lysine 76 (K76) mediated by GCN5L1 (General Control of Amino-Acid Synthesis, yeast homolog-like 1) inhibits the binding of TFAM to the mitochondrial transporter TOM70, resulting in reduced TFAM import into mitochondria and mitochondrial biogenesis (20). In contrast to these results, another work shows that increasing the acetylation levels of TFAM does not alter its binding to mtDNA, while significantly reducing TFAM-mediated DNA unwinding capacity (21). Although the role of TFAM acetylation is still debated, a growing body of evidence shows that PMTs in the C-tail of TFAM are critical for the recruitment and positioning of POLRMT (22) and TFBM2, leading to mitochondrial gene transcription (18).

Sirtuins (Sirts) are an evolutionarily conserved family of class III histone deacetylases that require NAD^+^ as a cofactor (23). Among these Sirt3, 4 and 5 have been found in mitochondria (24–26). However, only Sirt3 has been shown to have deacetylation activity, whereas the role of Sirt4 is that of ADP-ribosylase and lipoamidase, and Sirt5 is involved in succinylation, malonylation, and glutarylation (27). Interestingly, one of the targets of Sirt3 in the mitochondrion is TFAM (28) whose deacetylation results in increased binding to mtDNA, thus repressing gene transcription (28). Similarly, ERK2-mediated phosphorylation of TFAM in serine 177 (29) increases the binding of TFAM to mtDNA, resulting in suppression of transcription (19, 29). In addition, recent work shows that protein kinase A (PKA) regulates the phosphorylation of TFAM in serine 55 and promotes its degradation (30, 31).

In the nucleus, gene expression is not only modulated by PTMs of histones, but it is also regulated by the methylation in 5th position of cytosine (5 mC) in cytosine-guanine dinucleotide (CpG) at the level of regulatory sequences. Specifically, an increase in DNA methylation triggers the inhibition of transcription. The involvement and amount of methylated mtDNA are still debated. Some works report that methylation is a specific feature of genomic DNA that is not shared with mtDNA (32, 33), while other studies show the presence of methylation also at the level of mtDNA (34, 35, 36). Interestingly, in mtDNA, methylation of gene promoters has been observed at non-CpG sites (34). Although DNA methyltransferases DNMT1, DNMT3a and DNMT3b were shown to be active in mitochondria, their repression does not affect the mtDNA methylome (34). In mitochondrial DNA, the main methylation found is at the level of adenine (6 mA) at adenine-thymine dinucleotides (ApT) (37). In addition, accumulation of eukaryotic methyltransferase 4 MTA70 (METTL4) has been observed in the mitochondrial matrix (38), and knock-out of METTL4 results in a 6 mA decrease in mtDNA (38). Similar to nuclear DNA, in mitochondria 6 mA attenuates the binding of TFAM to transcription factors (38), suggesting the homology of the role of methylation in gene expression. In the nucleus, demethylation of 6 mA is driven by AlkB homolog 1 (ALKBH1) and ALKBH4 (39). However, there is currently no information on the mechanisms of 6 mA demethylation in mtDNA.

Another branch of epigenetic regulation is the role of noncoding RNAs (ncRNAs), a class of ribonucleic acid sequences that do not carry protein translation information but are involved in gene and protein regulation (40). Based on their length, ncRNAs are divided into long non-coding RNAs (lncRNAs), which are longer than 200 nts, and small ncRNAs (sncRNAs), which are shorter than 200 nts and include miRNA, cirRNA, and piRNA (40). Several roles have been established for lncRNAs: regulation of chromatin structure by interacting with histones, repression or activation of gene expression through hybridization with genomic DNA, regulation of splicing, stabilization of messenger RNAs (mRNAs), and sponging of miRNAs (41). On the other hand, miRNAs are known to interfere with the translation mechanism due to their complementarity with the 3′-untranslated region (3′UTR) of mRNAs (42).

Interestingly, metastasis-associated lung adenocarcinoma transcript 1 (MALAT1) was found in the mitochondrial matrix where it interacts with mtDNA in several regions (43). Specifically, this interaction represses the transcription of cytochrome C oxidase II (COX2), NADH: Ubiquinone oxidoreductase core subunit 3 (ND3) and cytochrome B (CYTB) genes encoded at the mitochondrial level (43). In addition, downregulation of MALAT1 in mitochondria leads to altered mtDNA copy number, mitophagy, apoptosis, and abnormalities in ATP production (43).

The RNA component of the RNase MRP ribonucleoprotein (RNP) complex (RMRP) lncRNA has also been found in the mitochondrial matrix where it acts as a primer for mtDNA replication (44). Furthermore, RMRP associates with nucleoporins involved in the export of small nuclear RNAs, highlighting its interaction between nuclear and mitochondrial epigenetic regulation (44).

Recent work reports the presence of mitochondria-specific miRNAs, called MitomiRs, which are subsequently transported into the cytosol to inhibit their target mRNAs or interact with mitochondrial proteins (45). One example is MitomiR-2392, which reduces the expression of mt-ND2, mt-ND4, mt-ND5, mt-CYTB, and mt-COX1 genes through interaction with Argonaute RISC Catalytic Component 2 (AGO2), which is present in the mitochondrial matrix (46). Dysregulation of mitomiR-2392 leads to altered cellular metabolism with decreased oxidative phosphorylation and increased glycolysis (46). Due to AGO2′s dual localization (cytosol and mitochondrial matrix) and ability to bind RNA, it has been proposed as an importer of miRNAs (47). Indeed, many miRNAs synthesized from genomic DNA have been found in mitochondria where they inhibit their targets. One example is miR-181c, which regulates levels of the mitochondrial protein mt-COX1 (48). Interestingly, overexpression of miR-181c did not change mt-COX1 mRNA but significantly decreased mt-COX1 protein, suggesting that miR-181c is primarily a translational regulator of mt-COX1 (48). In addition, overexpression of miR-181c results in increased mt-COX2 mRNA and protein content, with an increase in both mitochondrial respiration and reactive oxygen species generation, causing mitochondrial dysfunction (48). Similarly, human miR-198 and miR-765 have also been found in mitochondria, but their targets have not yet been established (47).

**Figure 1:**
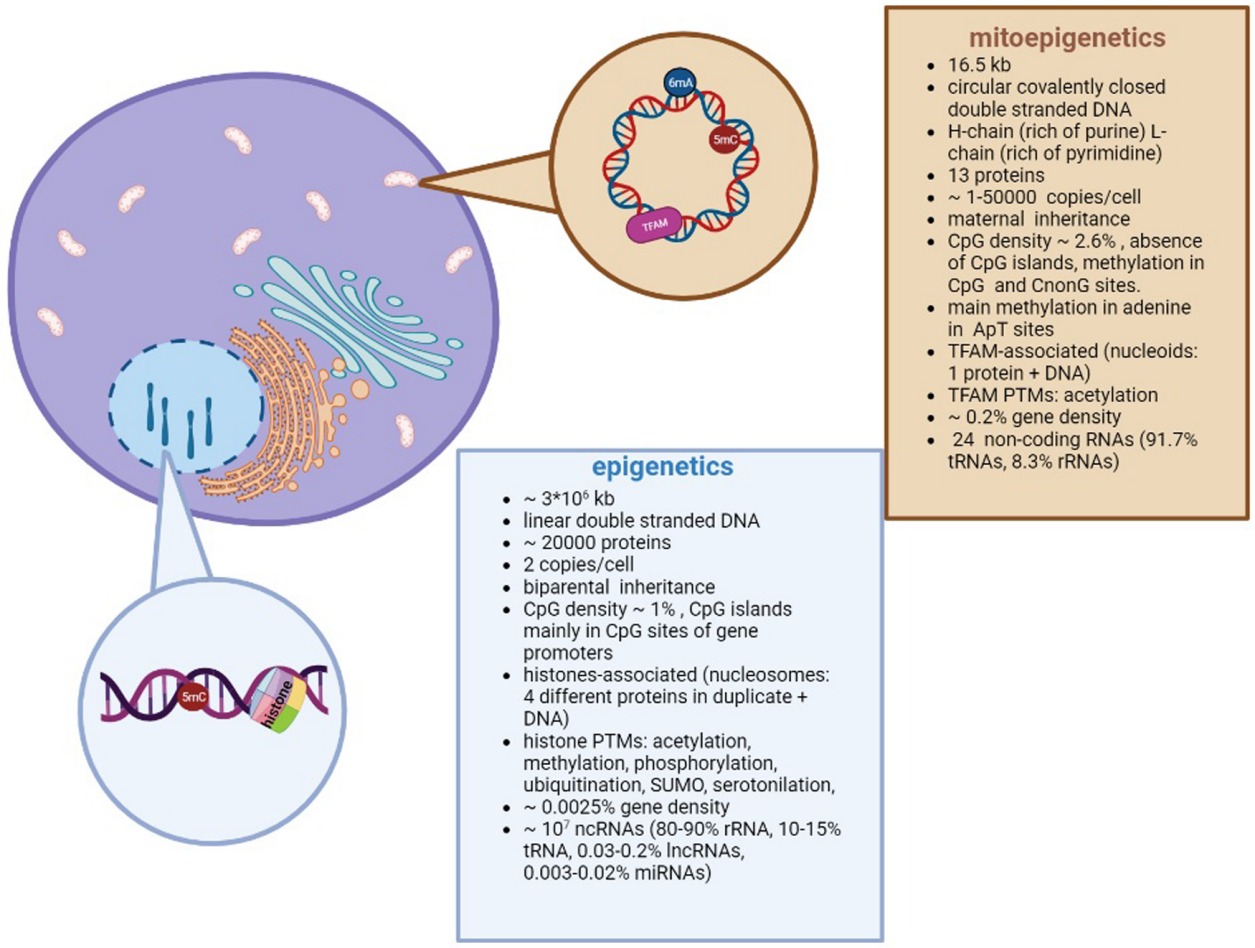
Differences between nuclear and mitochondrial Epigenetics. Despite different DNA structures (circular vs linear) and genome organizations (i.e. nucleoids vs nucleosomes; methylation in adenosine vs cytosine), both types of epigenetics are aimed to regulate the gene expression.

A brief overview about the differences between epigenetics and mitoepigenetics is summarized in Figure 1.

## Mitoepigenetics in aging

The aging process leads to nuclear epigenetic alterations such as remodelling of chromatin structure caused by changes in DNA methylation and histone PTMs, telomere shortening, and modulation of ncRNAs espression (49, 50, 51).

### The effects of TFAM post-translational modifications

TFAM plays an essential role in maintenance, expression, and organization of mitochondrial DNA. Indeed, it is required for efficient promoter recognition by mitochondrial RNA polymerase and binds and induces significant conformational changes in mtDNA. In muscles of aged flies, TFAM and mtDNA have been found to form a more condensed structure than in juveniles (52) impeding access to the mitochondrial transcriptomic machinery with a subsequent reduction of gene expression. In detail, from 1 day of age to 12 weeks of age, the size of nucleoids decreases while their number increases (52). Under normal conditions, overexpression of TFAM leads to the reduction of Drosophila lifespan, whereas in the presence of 1% H2O2, flies that up-regulate TFAM resist the environment more, revealing that TFAM is a central factor under severe oxidative stress and mitochondria maintenance (53). Despite the lack of research on TFAM and aging in mammals, the repression of mitochondrial gene transcription in the presence of a high level of TFAM protein is well known (54) suggesting a role of TFAM in aging.

Recently, it has been observed that DNA methyltransferase 3 alpha (DNMT3A) and ten-even-transposon 2 (TET2) regulates the TFAM expression in macrophages (55). Specifically, decreased DNMT3A and/or TET2 results in downregulation of TFAM with subsequent cytosolic mtDNA release (55). The presence of mtDNA in the cytosol is responsible for the activation of cyclic GMP–AMP synthase (cGAS) signalling that triggers interferon alpha (*IFN*α) production with subsequent increase in inflammation (55).

Notably, in aging is well known the increasing of inflammation state (named inflammaging) which involved several cytokines included IFN (56). This finding suggests that TFAM migh contribute to the burst of inflammation observed in aging. in aging is well known the increasing of inflammation state (named inflammaging) which involved several cytokines included IFN (56). In young mice, lymphocyte-specific Tfam knockout recapitulated the characteristics of mitochondrial dysfunction that occurs in aged (22-month-old) wild-type mice, and this mitochondrial decline is characterized by increased secretion of inflammatory cytokines, such as interferon-γ (IFN-γ) and tumor necrosis factor-α (TNF-α) (57).

It is well known that nuclear translocation of p53 protein is associated with increased aging due to its inhibition of PPARγ co-activator 1α (PGC1α) and PGC1β (58). It is interesting to see that in skeletal cells after endurance exercise, the presence of nuclear p53 is reduced while its presence is increased in mitochondria where it serves to positively modulate the activity of the mitochondrial transcription factor TFAM (59). Based on this evidence, physical activity in the elderly could also improve mitochondrial function through the p53-TFAM interaction.

### Methylation of mtDNA

Another epigenetic modification that occurs in aging is the global decrease in the level of methylation in genomic DNA, which cooperates with the modification of histones (60). Despite the global decrease in 5mC, it has been observed that some specific CpGs are particularly hypo- or hyper-methylated. This signature has been used to develop so-called “epigenetic clocks” that are able to specifically detect the biological age of individuals (61–65).

Although the most representative methylated base in mtDNA is adenine, recent work shows a correlation between aging and 5 mC methylation of mtDNA in the brain, where some 5 mCs (in this case both CpG and non-CpG) were found to be modulated (66). Unlike genomic DNA, aged mtDNA reveals a pattern of global hypermethylation (66). This feature is likely due to the loss of the ability of ten-eleven transposons (TETs) to eliminate methylation on cytosines, caused by altered metabolism in which the reduction of α-ketoglutarate (the cofactor of TETs) was observed (67). In recent work, a significant increase in DNA methylation levels was found according to age and the administration of the low-calorie diet. Particularly, the increase in methylation level represses genes involved in mitochondrial biogenesis, suggesting that mitochondrial dynamics is driven by age and diet (68). During aging, mitochondrial increase in reactive-oxygenated species (ROS) has been reported to modulate mtDNA (69, 70). Specifically, ROS-induced damage to mtDNA reveals impaired production or mutation of enzymes involved in oxidative phosphorylation, exacerbating mitochondrial dysfunction and enhanced cellular senescence (69).

### Mitochondrial non-coding RNAs

The correlation between miRNA and histone deacetylase has been demonstrated during aging. Specifically, miR-9 and miR-34a have been found to be up-regulated in aging and both target Sirt1 mRNA, which is found to be down-regulated contributing to enhanced gene expression (71). In addition, Sirt1 is also regulated by miR-181a, which is down-regulated during aging instead (72). Repression of miR-181a in the elderly has been associated with defects in T-cell activation (72). The lncRNAs are also modulated in aging. For example, H19 is down-regulated in senescent endothelial cells (73). Specifically, repression of H19 reflects increased phosphorylation of STAT3, which becomes active and transcribes p16 and p21 reflecting cell cycle inhibition (73). In addition, in cardiomyocytes (CM), lncRNA ENSMUST00000134285 has been observed to control apoptosis. In this case, in aged tissues, the expression of ENSMUST00000134285 is increased and inhibits MAPK11 activity, initiating the apoptotic process (74).

Modulation of ncRNAs has also been observed in aging, and the ncRNAome is tissue-specific. An example is miR-183-5p, miR-199b-5p, miR-205-5p, and miR-200b-3p, which are up-regulated in 3-month-old mice and are involved in the thymus regression process (75). Similarly, modulation of the lncRNAome during aging was found in the liver, where Meg3, Rian and Mirg were found to be up-regulated, revealing increased inflammation, repression of cell proliferation and metabolic changes (76). The lncRNA nuclear-enriched abundant transcript 1 (NEAT1) was found to be modulated in bone marrow mesenchymal stem cell (BMSC) senescence (77). Up-regulation of NEAT1 drives the transition from bone cells to fat cells by hindering mitochondrial function, while repression of NEAT1 reflects an increase in Sirt3 (77), which, as has been discussed previously, is responsible for deacetylating TFAM (28).

In conclusion, the ncRNAs that are transcribed by nuclear DNA and shifted to mitochondria have been found to modulate oxidative stress response, Sirt1-dependent deacetylation, inflammation, cell cycle and apoptosis confirming their involvement in mitochondrial homeostasis leading to aging process (Table 1).

**Table 1:** Overview of mitoepigenetic modifications in aging.

Target	Modification	Effects	Organism
TFAM	Condensed structure of nucleoids	Less contractility	*Drosophila* (wing muscle)
Mt-DNA			
Mt-DNA	Increase of 5 mC	• Repression of mitochondrial genes transcription • Impairment of oxidative stress response • Accelerated aging	Humans and mammals
mt-DNA	5 mC age-related in brain	• Mitochondrial brain-specific epigenetic clock	Humans
miR-181a	Down-regulated	• Repression of Sirt1 • Accelerated aging • Dysregulation of T-cell activation	Humans
miR-183-5p	Up-regulation	• Timus regression	Mice
miR-199b-3p			
miR-205-5p			
miR-200b-3p			
*Meg3*	Up-regulation	In liver: • Increase of inflammation • Proliferation arrest • Metabolic changes	Mice
*Rian*			
*Mirg*			
NEAT1	Up-regulation	• Impairment of mitochondrial function • Switching BMSCs to fat cell • Skeletal aging	Mice
H19	Down-regulated	• Increase of STAT3 phosphorylation¨ • Up-regulation pf p16 and p21 • Endothelial senescence	Humans and mammals
ENSMUST00000134285	Up-regulation	• Inhibition of MAPK11 • Cardiomyocytes apoptosis	Mammals

## Mitoepigenetics in cardiovascular diseases

During aging, the risk of developing cardiovascular disease (CVD) increases (78). On the other hand, various risk factors such as unhealthy dietary regimens, physical inactivity, smoking, and alcohol abuse play a major role in the development and acceleration of cardiovascular diseases (2, 79, 80). Heart failure (HF), coronary heart disease, rheumatic heart disease, and stroke are among the leading causes of death globally (17.9 million per year), and the involvement of epigenetics in the development of these diseases has been extensively studied (81, 82, 83, 84, 85, 86). However, growing evidence suggests the potential contribution of mitoepigenetics in the pathophysiology of CVD.

### Role of mtDNA methylation in CVDs

Modulation of the mtDNA methylation of the mt-COX2 gene has been observed in human cardiac mesenchymal stem cells (HMSCs) (87). In particular, senescent HMSCs reveal hypermethylation of the COX2 gene, which appears to be downregulated (87). On the other hand, overexpression of mt-COX2 and inhibition of DNMT1 by 5-aza-2′-deoxycytidine delay the senescence process (87). It has also been shown that, in the rat model, depletion of mt-COX2 reduces ATP and acetyl-CoA production. Consistently, the expression of genes related to mitochondrial oxidation, were downregulated, while glycolytic hexokinase 1 (HK1) was upregulated. These observations indicate that COX2-deficient rats develop hypertension, heart failure, and increased thrombotic events, probably as a result of dysregulation of cardiac energy metabolism (88).

Analysis of mtDNA methylation was also performed in platelets from CVD patients, where an increase in 5mC methylation was found in comparison with healthy controls (89). In particular, an increase in the methylation level of the mt-COX1/2/3 gene and mitochondrial leucine 1 (mt-TL1) tRNA was found (89). In line with these findings, in vascular smooth muscle cells (VSMCs) platelet-derived growth factor-BB (PDGF-BB) triggers the translocation of DNMT1 from the nucleus to the mitochondria, where mtDNA methylation increases resulting in suppression of gene expression (90). These increased levels of methylation are also associated with mitochondrial dysfunction, altered contractility of VSMCs, and aberrant cell growth (90).

Mitoepigenetics also seems to play an important role in CAD. Indeed, comparing mtDNA methylation levels of peripheral blood leukocytes from patients with stable coronary artery disease (SCAD) and acute coronary syndrome (ACS), a lower level of global 5 mC mtDNA was found in patients with ACS. In line with these findings, hypermethylation of the D-loop region was also detected in these patients, which is associated with reduced mtDNA synthesis (91)⁠.

### Regulation of TFAM and nucleoids in the CVDs

In myocardial infarction, alteration of physiological functions of mitochondria leads to modification of proteins and lipids and inhibition of energy production, contractile capacity, cell necrosis, or apoptosis. In the myocardium, oxidative stress caused by ischemia/reperfusion (I/R) injury triggers nuclear translocation of nuclear respiratory factor 1 (NRF1) and upregulation of PPARG coactivator 1 alpha (PGC-1α), which increase TFAM transcription and contribute to mitochondrial biogenesis and repair, acting as a compensatory mechanism(92). Interestingly, TFAM depletion reflects the loss of cellular ability to respond to I/R damage. In CMs after the decrease in oxygen level, the amount of TFAM increases as a compensatory mechanism. Thereafter, its level progressively decreases revealing the increase in ROS production and calcium mismanagement. This phenomenon has been particularly observed in the later stages of HF. Therefore, restoring TFAM levels by increasing mitochondrial biogenesis and reducing ROS production could protect cardiomyocytes from the oxidative damage of mtDNA induced by I/R-injury (93).

Mitochondrial dysfunction is also a feature of hypertension that leads to increase vascular oxidative stress. In particular, Sirt3 has been found to play a key role in maintaining endothelium function (94). In the aortas of hypertensive mice, Sirt3 down-regulation was found to result in worsened blood pressure, vascular relaxation, superoxide and nitric oxide production, as well as increased hypoxia-induced factor 1 alpha (HIF1α), pro-inflammatory gene expression as well as vascular permeability (94). In addition, reduction of Sirt3 leads to increased acetylation of superoxide dismutase 2 (SOD2), resulting in loss of its antioxidant activity (94).

Consistently, beneficial effects of Sirt3 have also been observed (94). In a rat model, activation of the AMP-activated protein kinase (AMPK)-PGC1α-Sirt3 signaling pathway was observed (94). Specifically, PGC1α increases the expression of Sirt3, which in turn deacetylates NRF1, the transcription factor responsible for TFAM transcription (94). On the other hand, Sirt3 also acts directly on TFAM allowing its translocation into mitochondria and subsequently transcription of mitochondrial genes.

Activation of this pathway, thus on the one hand enhances mitochondrial biogenesis and function while on the other hand reduces ROS-dependent cellular stress (94). Interestingly, this pathway can be stimulated by melatonin administration through AMPK activation (94).

Consistently, beneficial effects of Sirt3 have also been observed in myocardial I/R injury (95). In a rat model of I/R, activation of the AMP-activated protein kinase (AMPK)-PGC1α-Sirt3 signalling pathway has been observed (95).. Specifically, PGC1α increases the expression of Sirt3 which in turn deacetylates NRF1 the transcription factor responsible for TFAM transcription (95). Moreover, Sirt3 also acts directly to TFAM allowing its translocation into mitochondria and subsequently transcription of mitochondrial genes. As a result,the biogenesis and function are gained and ROS-dependent cellular stress is reduced (95). Interestingly, this pathway can be stimulated by melatonin administration, which acts by activating AMPK (95). Similarly, the PGC1α-Sirt3-TFAM/NRF1 pathway was also found to be repressed in a rat model of isoprotenerol-induced HF (96). Of note, administration of perindopril improves cardiac function through SIRT3 and PGC1α signalling pathway activation (96).

### Mitomirs and mitochondrial lncRNAs modulation in CVDs

In CVDs, several mitomiRs have been found to be involved in the pathogenesis. An example is miR-181c, a nuclear-encoded miRNA, which is translocated from nucleus to mitochondria and targets mt-COX1 mRNA (97)⁠. To mimic the HF condition, miR-181c has been up-regulated targeting nanoparticles to heart (97)⁠. The miR-181c- treated rats revealed a significant decrease in left ventricular fractional shortening and markedly lower ejection fraction (97). MiR-181c has also been found to regulate calcium uptake in cardiomyocytes (98)⁠. The miR-181c loss can protect the heart from I/R injury by modulating calcium transport through the upregulation of mitochondrial calcium uptake 1 (MICU1) (98)⁠. In fact, in miR-181c^−/−^ mouse model, the molecular mechanism found is the involvement of mt-COX1 which up-regulates the transcription factor specificity protein 1 (Sp1) that in turn triggers the expression of MICU1 (98).

Several miRNAs are involved in the pathogenesis of CVD. One example is miR-181c, a nuclear-encoded miRNA that is translocated from the nucleus to the mitochondria and targets mt-COX1 mRNA (97). To recapitulate an HF phenotype, miR-181c was up-regulated by targeting nanoparticles to the heart (97). Rats treated with miR-181c revealed a significant decrease in fractional shortening and ejection fraction (97). It was also found that miR-181c regulates calcium uptake in cardiomyocytes (98). Thus, suppression of miR-181c may protect the heart from I/R injury by modulating calcium transport through upregulation of mitochondrial calcium uptake 1 (MICU1) (98). Indeed, in the miR-181c^−/−^ mouse model, the molecular mechanism found is the involvement of mt-COX1 regulating the transcription factor specificity protein 1 which in turn triggers the expression of MICU1 preserving Ca^2+^ uptake in CMs after I/R injury (98). Interestingly, in the human heart, up-regulation of miR-181c leads to ventricular septal defects (99). In this condition, the molecular mechanism of miR181c involves inhibition of bone morphogenetic protein type 2 receptor (BMPR2) (99), which is critical for septal formation and valvulogenesis (100).

Small RNA-seq performed in mitochondria isolated from mouse failing hearts, showed that mitochondria-enriched microRNAs in HF were associated with energy metabolism and oxidative stress pathway (101). Little is known about these mitomiRs in CVDs. However, miR-696 has been observed to down-regulate PGC1α with subsequent impairment of mitochondrial function in skeletal muscle (102)⁠. An analogous mechanism might occur also during I/R injury in CMs, however no information is currently available.

On the other hand, lncRNA growth arrest specific 5 (GAS5) is known to sponge miR-532-5p and is up-regulated during I/R (103). Specifically, this lncRNA is increased in I/R injury and is responsible for phosphoinositide-3-kinase (PI3K)/protein kinase B (AKT) downregulation, thus resulting in apoptosis (103). Overexpression of GAS5 lncRNA during ischemic injury prevents the development of an adverse cardiac phenotype (103). Similarly, during stroke, miR-532-5p expression is strongly reduced and is associated with increased infarct area, neuronal apoptosis, and worsening neurological score (104). The up-regulation of miR-532-5p enhances the PI3K/AKT pathway that increases cell viability by attenuating neurological damage (104).

In hypoxia, the potentiation of miR-210 and its involvement in CVD is well known (105). Iron-sulfur cluster assembly proteins 1 and 2 (ISCU1/2) have been found to be a target of miR-210 in mitochondria (106). Increased miR-210, under oxygen-deficient conditions, reduces the level of ISCU1/2, which is involved in iron-sulphur clusters for electron transport and mitochondrial redox reactions (106). Consequently, downregulation of ISCU1/2 impairs metabolism and cell survival.

MitomiRs are also involved in cardiac hypertrophy. MiR-485-5p has been found repressed in mouse hypertrophic heart where it interacts with the mitochondrial anchored protein ligase (MAPL). In turn, the downregulation of MAPL reveals the over-expression of the mitochondrial fusion protein 2 (MFN2) which induces the mitochondrial fission (107)⁠. In hypertrophic hearts, miR-485-5p up-regulation was shown to reduce LV wall thickness (107)⁠. miR-485-5p is also regulated by metastasis-associated lung adenocarcinoma transcript 1 (MALAT1) (108)⁠ that is over-expressed in hypoxic condition (109)⁠. In CVD patients, high level of circulating MALAT1 has been observed in association with inflammation and hypoxia (110)⁠. Notably, in hepatocellular carcinoma it has been found that MALAT1 binds the mtDNA modifying the mitochondrial structure, reducing oxidative phosphorylation and ATP production, decreasing mtDNA copy number, and activating the apoptosis (43)⁠. Despite the increase of MALAT1 in CVDs, no work reports the modulation of mitochondrial gene expression upon MALAT1-mtDNA interaction.

**Figure 2:**
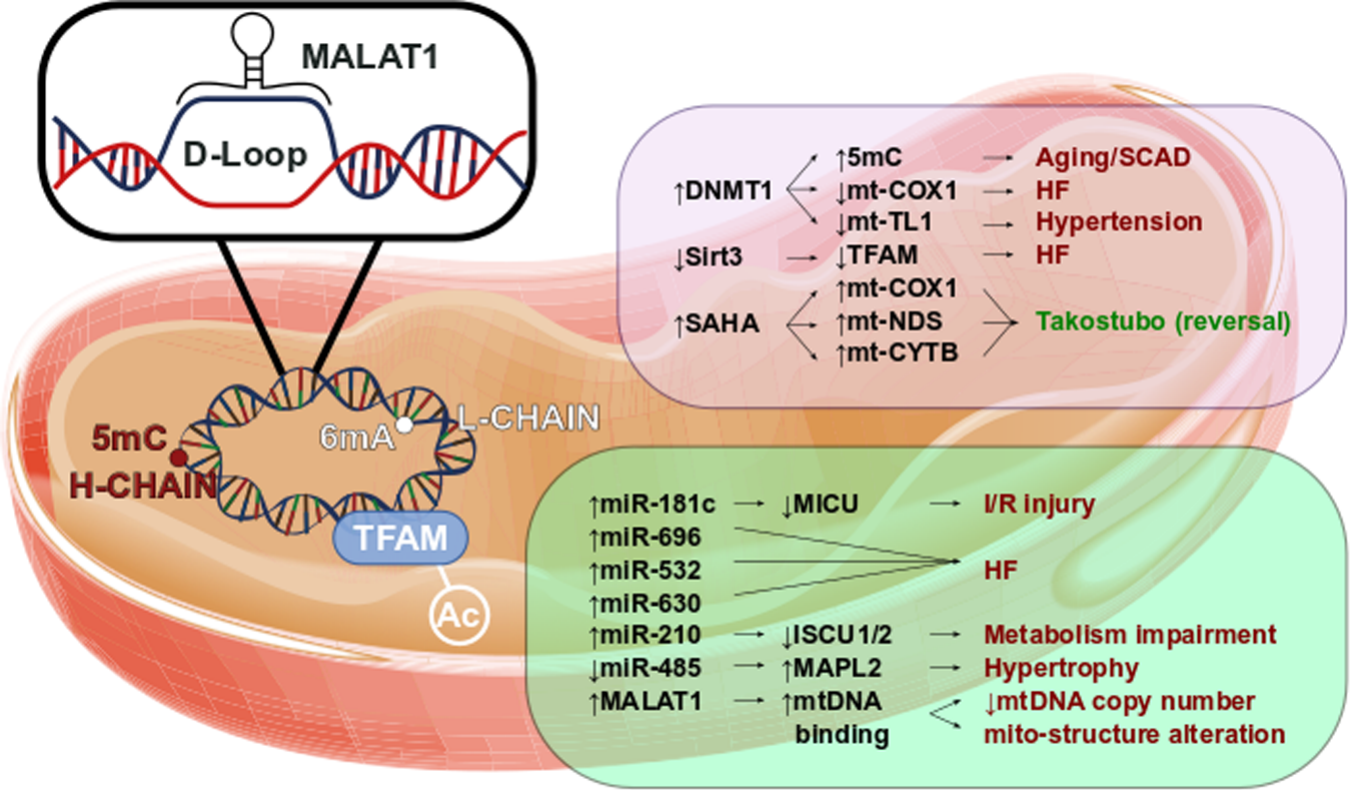
Mitoepigenetics of cardiovascular diseases. Several epigenetic modifications might occur in mitochondrial matrix. The mt-DNA methylation occurs mainly in Adenine of L-chain. However, the 5mC in H-chain has been found to have a regulatory function. In fact, the increase of 5mC has been observed in aged mitochondria as well in mitochondria leucocyte of SCAD patients. In parallel, high level of 5mC in mt-COXQ and in mt-TL1 promoters triggers the HF and hypertension. Additionally, the retains of TFAM acetylation, caused by the decrease of Sirt3 activity, has been observed in HF. Little is known of SAHA effects in mitochondrial regulation, however we might suppose that SAHA modulates the expression of mt-COX1, mt-NDS, and mt-CYTB contributing to Takotsubo disease reversion. Pivotal roles have also been observed in mitochondrial ncRNA regulation. Higher level of miR-181c is leaded to the worsening of I/R injury as well the up-regulation of miR-696, miR-532 and miR-630 triggers the HF. The impairment of metabolism has been associated to the increase of miR-210 through the repression of ISCU1/2. The down-regulation of mitochondrial miR-485 increases MAPL2 expression which stimulates the hypertrophic growth. lncRNAs are involved in CVD too. In fact, MALAT1 has been found to bind the D-Loop of mt-DNA inhibiting the synthesis of new mt-DNA. The reduction of mt-DNA copy number reflects the alteration of mitochondrial structure and the impairment of the oxidative phosphorylation.

Figure 2 reports a brief overview of mitochondrial epigenetics in cardiovascular diseases.

## Future perspectives of mitoepigenetics in treatment

Mitoepigenetics is arising as a new marker of aging (51)⁠ and CVDs ⁠(13). However, therapeutic interventions, that specifically target a mitoepigenetic factor, are difficult to develop due to the strict correlation to the genomic regulation. An example is represented by DNMT1 which methylates nuclear DNA and it translocates into mitochondria where acts on the mtDNA (36, 66, 90, 111)⁠. The inhibition of DNMT1 with 5-aza-2′-deoxycytidine has been observed to decrease the amount of 5 mC in mitochondria associated to the improvement of mt-COX2 expression (87). However, the hypomethylation has been observed in genomic DNA in response to 5-aza-2′-deoxycytidine administration which induces genomic instability and tumor growth (112)⁠. Similarly, the inhibition of HDACs by SAHA reverts the heart remodelling through increased H3 acetylation (113). However, no data are available on SAHA effects on mitochondrial deacetylases that regulate TFAM. Analogously, several miRNAs have shown an active interplay between cytosol and mitochondrial matrix (45). Hence, modulation of their activity within a specific cellular compartment (without altering the other) remains challenging.

Physical activity is known to influence nuclear epigenetics by increasing lifespan through activation of sirtuins, including the mitochondrial protein SIRT3 (114). During the exercise, there is upregulation of mitochondrial TFAM as well as of protein involved in beta-oxidation, Krebs cycle and electron transport chain. This results in improvement of ROS clearance, enhancing the fusion process between functional organelles and the removal of dysfunctional mitochondria reduce thus reducing the onset of senescence (115). Interestingly, in the elderly population (65 ± 7 years), exercise alters the mtDNA methylome in skeletal muscle making it similar to that of younger men (116).

In conclusion, mitoepigenetics and epigentetics seem to cooperate for the cellular homeostasis. In fact, both systems share proteins (i.e., DNMT1) and ncRNAs (i.e., MALAT1, miR485 which are synthetized in the nucleus and shuttled in mitochondia and an alteration of one mechanism might modulate the other (43, 87, 107). As an example, Dunham-Snary (112, 116) demonstrated that mtDNA can modulate nuclear gene expression in adipose tissue (117).

Application of machine learning to identify novel cardiac biomarkers reveals that total nuclear methylation and methylation in a specific CpG island of TFAM were the best diagnostic measures related to diabetes progression (118). Also this study support the theory that epigenetics and mitoepigenetics are two processes interconnected.
